# Recycled high-density polyethylene plastic reinforced with ilmenite as a sustainable radiation shielding material

**DOI:** 10.1039/d3ra03757f

**Published:** 2023-07-10

**Authors:** M. I. A. Abdel Maksoud, Said M. Kassem, A. H. Ashour, A. S. Awed

**Affiliations:** a Radiation Physics Department, National Center for Radiation Research and Technology (NCRRT), Egyptian Atomic Energy Authority (EAEA) Cairo Egypt muhamadmqsod@gmail.com; b Radiation Protection and Dosimetry Department, National Center for Radiation Research and Technology (NCRRT), Egyptian Atomic Energy Authority (EAEA) Cairo Egypt; c Higher Institute for Engineering and Technology at Manzala Egypt

## Abstract

In this work, recycled high-density polyethylene plastic (r-HDPE) reinforced with ilmenite mineral (Ilm) in different ratios (0, 15, 30, and 45 wt%) as a sustainable and flexible radiation shielding material was manufactured using the melt blending method. XRD patterns and FTIR spectra demonstrated that the polymer composite sheets were successfully developed. The morphology and elemental composition were addressed using SEM images and EDX spectra. Moreover, the mechanical characteristics of the prepared sheets were also studied. The gamma-ray attenuation characteristics for established r-HDPE + *x*% Ilm composite sheets were theoretically computed between 0.015 and 15 MeV using Phy-X/PSD software. Also, the mass attenuation coefficients have been compared to their values by the WinXCOM program. It is also shown that the shielding performance of the r-HDPE + 45% Ilm composite sheet is significantly greater than that of r-HDPE. As a result, the ilmenite-incorporated recycled high-density polyethylene sheets are suited for medical and industrial radiation shielding applications.

## Abstract 2

In this work, recycled high-density polyethylene plastic (r-HDPE) reinforced with ilmenite mineral (Ilm) (0, 15, 30, and 45 wt%) as a sustainable and flexible radiation shielding material was manufactured using the melt blending method.

## Graphical abstract

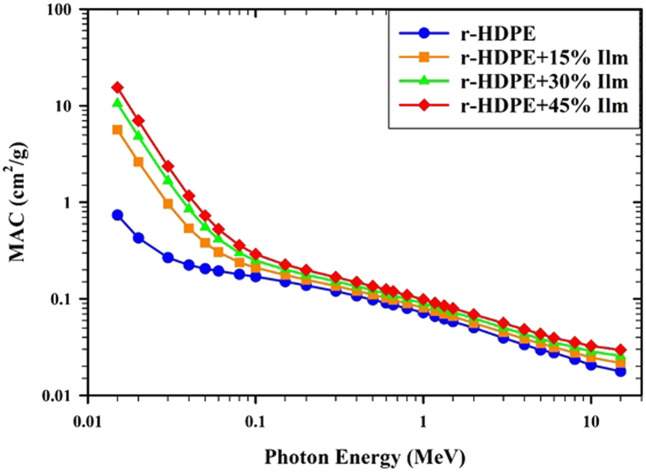

## 1. Introduction

Plastic recycling has gained significant attention as a waste-management approach in recent years due to its potential for reducing adverse environmental effects and energy and raw material consumption in polymer manufacturing. This aligns with the principles of a circular economy strategy, which aims to promote sustainability.^1^ The interest in radiation shielding materials has led to a desire for enhanced plastic composites that meet demanding requirements, including mechanical strength. Further, it has been proposed that polymeric materials could serve as effective shields for gamma- and X-rays in the event enriched with heavy minerals or metals due to their effective attenuation characteristics. Several economically feasible plastics can be blended with plastic waste, including polyethylene resins, polypropylene, silicone rubber, polyvinyl alcohol/chloride (PVA/C), and polystyrene.^2–5^

High-density polyethylene HDPE has been the world's most extensively developed synthetic plastic polymer in recent decades, having a broad range of uses owing to its cheap cost, flexibility, lightweight, eco-friendly, excellent mechanical properties, and anti-corrosion properties. Due to its low melting point, polyethylene may not be suitable for space vehicle panels or industrial applications, but it may be suitable for protecting workers in industries that utilize energy radiation. This type of filler may be used in enhancing the physicomechanical and the radiation attenuation parameter characteristics of HDPE polymers.^6–8^

Produced recycled plastic can be utilized to manufacture novel products, including automobile parts, floor coverings, floral planters, park benches, tables for picnics, waste paper receptacles, crates, plastic timber, and wood–plastic composites, which will offer recycled plastics with additional market value. For applications such as space, industry, and radiation facilities, composite materials derived from post-consumer recycled thermoplastics can be used to fabricate sustainable and cost-effective radiation shielding materials.^6,9,10^ The developed shielding materials are cost-effective and environmentally friendly plastic waste based on r-HDPE, PbO NPs, and bulk powder.^6^

The strategic selection of dense mineral or metallic additives, combined with gamma and X-ray radiation, can provide effective shielding, low cost, and easy fabrication. Incorporation of the ilmenite mineral is recognized for its high attenuating properties and cost-effectively due to its existence in a wide range of igneous and metamorphic rocks. Abdo *et al.*^11^ have investigated the potential application of an ilmenite/epoxy composite to attenuate neutrons and gamma rays. It was employed as an aggregate in producing heavy ilmenite/epoxy injecting mortar. This procedure was performed to address the issue of cracks that develop in concrete biological shields and to provide effective attenuation of neutrons and gamma rays.

This investigation aims, for the first time, to develop cost-effective and sustainable plastic waste materials based on high-density polyethylene (r-HDPE) containing varying concentrations of ilmenite (Ilm) to develop innovative radiation shielding composite sheets that exhibit beneficial γ-ray shielding features using Phy-X/PSD software. Also, the structural and mechanical characteristics of the r-HDPE + *x*% Ilm composite sheets have been addressed. In addition, the mass attenuation coefficients of the composite sheets have been compared to their theoretical values by NIST's WinXCOM program.

## 2. Experimental procedure

### 2.1. Materials

Recycled HDPE PE100 granules produced by SABIC company, with a density of 0.945 g cm^−3^. Elnasr Mining Company recovered ilmenite ores (Ilm) as fine and coarse aggregates from the Red Sea in Egypt. Ilmenite is a mineral composed chiefly of iron and titanium oxides in concentrations ranging from 20 to 35 wt% and 35 to 65 wt%, respectively. Manganese and magnesium may dissolve into the ilmenite structure, but silicon and aluminum are distinct oxides. The nominal particle sizes were at the micro range (<100 μm) and were separated by hand sieving.

### 2.2. Preparation of HDPE modified with ilmenite composite sheets

**Fig. 1:**
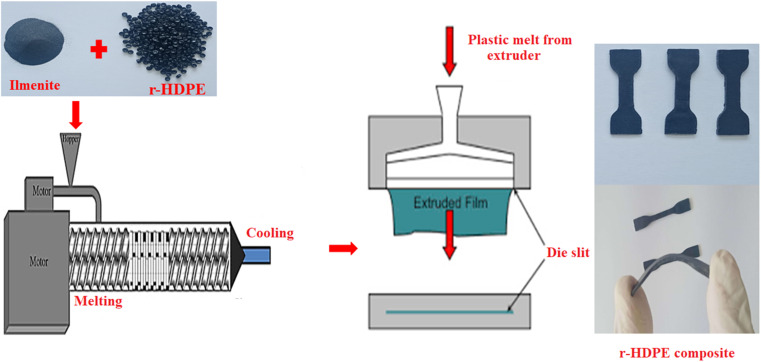
Schematic diagram of r-HDPE and r-HDPE + *x*% Ilm composite sheets preparation.

Recycled-high-density polyethylene (r-HDPE) pellets have been loaded to the laboratory plastic mixer (model PL-2100) Brabender plastic mixer with two screw blades type 350-S. Once the HDPE pellets were fully melted, quantities of ilmenite additives in specified ratios by weight (0, 15, 30, and 45 wt%) were supplied *via* the top hole into the heated mixer bowl at 160 °C. The r-HDPE and ilmenite powder were thoroughly mixed employing the double-helical mixer at 20 rpm for 20 minutes. After thoroughly mixing, the composites are inserted into aluminum molds and squeezed for 5 minutes in a hot press at about 160 °C before being cooled, as illustrated in Fig. 1.

### 2.3. Characterization techniques

Shimadzu XRD-6000 diffractometer with Cu target was used to measure the XRD patterns of r-HDPE + *x*% Ilm composite sheets. The EDAX spectra, obtained using a JEOL JSM-5600 LV instrument from Japan, were utilized to examine composite sheets of r-HDPE and *x*% Ilm. The functional groups of the examined sheets were validated using Fourier transform infrared (FT-IR) spectroscopy, utilizing the NICOLET iS10 model instrument, within the 4000–350 cm^−1^. High-resolution scanning electron microscopy (SEM), JEOL JSM-5600 LV, Japan, was used to gain information on the morphology of r-HDPE + *x*% Ilm composite sheets. The mechanical characteristics of r-HDPE + *x*% Ilm composite sheets, such as tensile strength and elongation at break, were measured using the tensile testing machine Qchida computerized testing machine, Dongguan Haida Equipment Co. Ltd, China. The mechanical parameters were determined utilizing dumbbell-shaped test pieces at a crosshead speed of 500 mm min^−1^ at 25 °C. Tensile strength and elongation at break were measured using the ISO 527-2 and ASTM D 412a-98 standards. In triplets, the mechanical characteristics were investigated.^12^

Using the following equation, the Archimedes rule was applied to determine the density of toluene-soaked r-HDPE composites:1
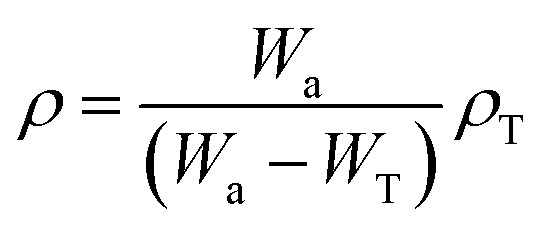
where, *W*_a_ would be the weight of the r-HDPE composite sheet when it is in the air, *W*_T_ is the weight of the composite sheet in toluene liquid, and *ρ*_T_ is the density of toluene liquid (0.86 g cm^−3^ at room temperature).

### 2.4. Determination of radiation-attenuating coefficients

Phy-X/PSD results from extensive effort recently completed by Şakar and his team^13^ to create an easy-to-use program that can produce various radiation protection factors. It is accessible online at (https://phy-x.net/PSD) and may help researchers and shielding engineers investigate and provide data on photon attenuation using various systems. Additionally, using NIST's WinXCOM computer program, the mass attenuation coefficients have been compared to their theoretical values by Phy-X/PSD.^14,15^

Calculating the mass attenuation coefficient MAC (*μ*_m_) entails dividing the linear attenuation coefficient (*μ*) by the density *ρ* of the r-HDPE + *x*% Ilm composite sheets;^13^2
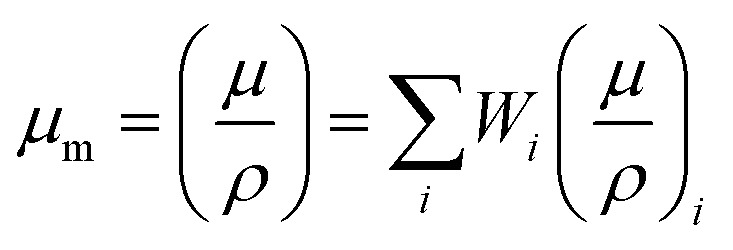
In which *W*_*i*_ symbolizes the weight fraction of the *i*^th^ element in the sample.

The half-value layer (HVL) defines the sample's thickness, decreasing the radiation's intensity by half. The tenth-value layer (TVL) refers to the mean amount of substance required to attenuate 90% of the entire radiation, reducing the initial intensity to one-tenth of its original value. Furthermore, the mean free path (MFP) is the average distance a photon travels between two subsequent interactions. The HVL, TVL, and MFP are provided in the formulae in ref. ^16^ and ^17^;

3 HVL = ln(2)/*μ*

4 TVL = ln(10)/*μ*

5 MFP = 1/*μ*

## 3. Results and discussion

### 3.1. Structural studies

**Fig. 2:**
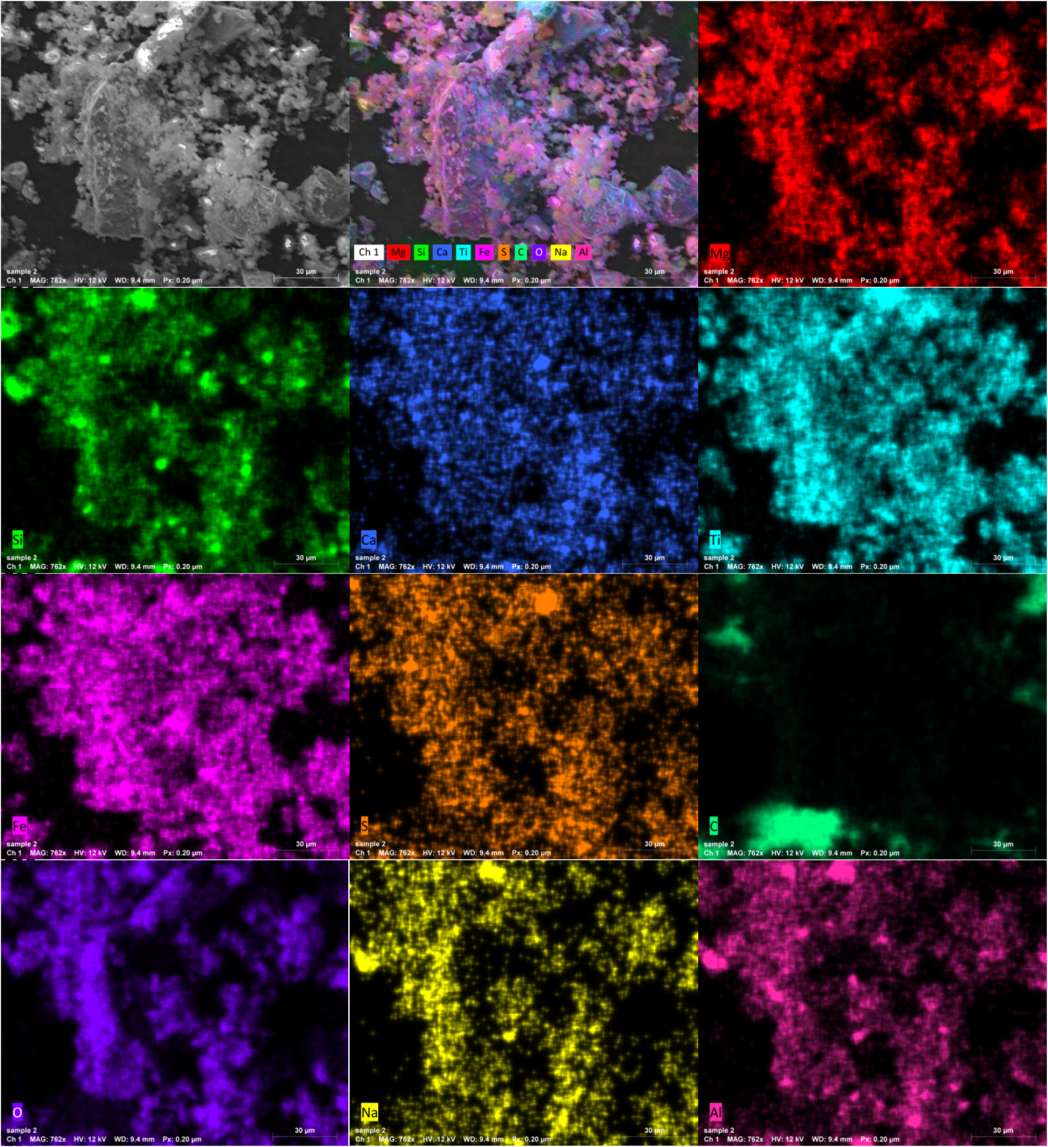
SEM and elemental mapping images of ilmenite powder.

Also, the surface morphology of Ilm powder was presented in Fig. 2. SEM image shows that the ilmenite has agglomerated particles with fine sizes. Also, the mapping images revealed that the fundamental elements were distributed uniformly over the ilmenite powder sample. Further, Fig. 2 demonstrate the presence of Fe, Ti, Si, Mg, Al, Ca, Na, S, C, and O elements, which refer to natural ilmenite mineral.

**Fig. 3:**
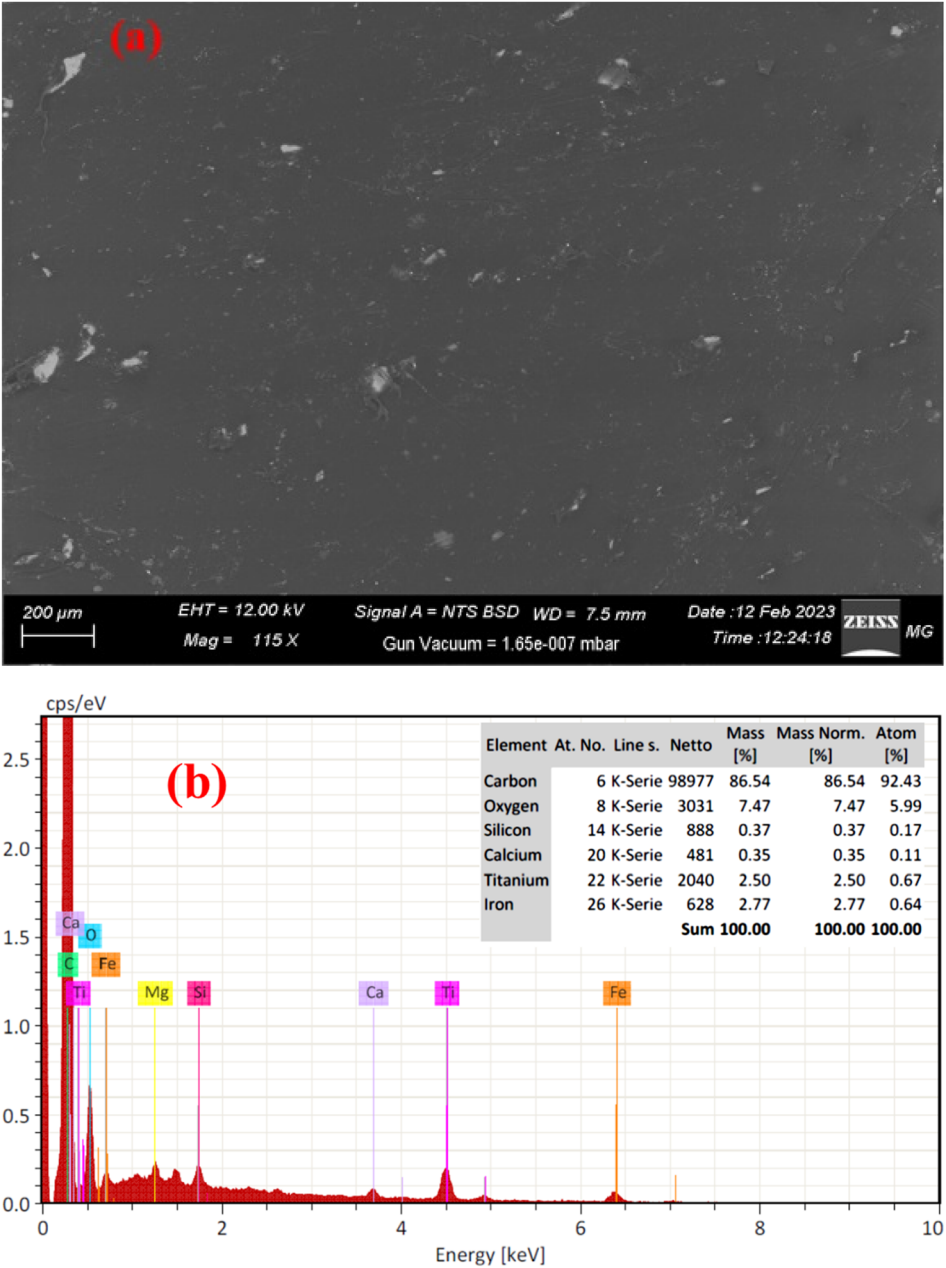
(a) SEM images, and (b) EDX of r-HDPE + 45% Ilm composite sheet.

Fig. 3(a) illustrates the surface morphology characteristics of the composite sheets composed of r-HDPE and *x*% Ilm filler. Fig. 3(a) shows that the addition of ilmenite powder to the r-HDPE + 45% Ilm composite sheet results in a quasi-spherical structure in the SEM microstructure. Despite a few aggregates, ilmenite can be seen dispersed throughout the r-HDPE matrix. Signals attributable to ilmenite contents (Fe, Ti, Si, Mg, Al, and Ca) can be seen in the EDX spectra and elemental composition of the r-HDPE + 45% Ilm composite sheet shown in Fig. 3(b). This suggests that ilmenite been successfully incorporated into the r-HDPE matrix.

**Fig. 4:**
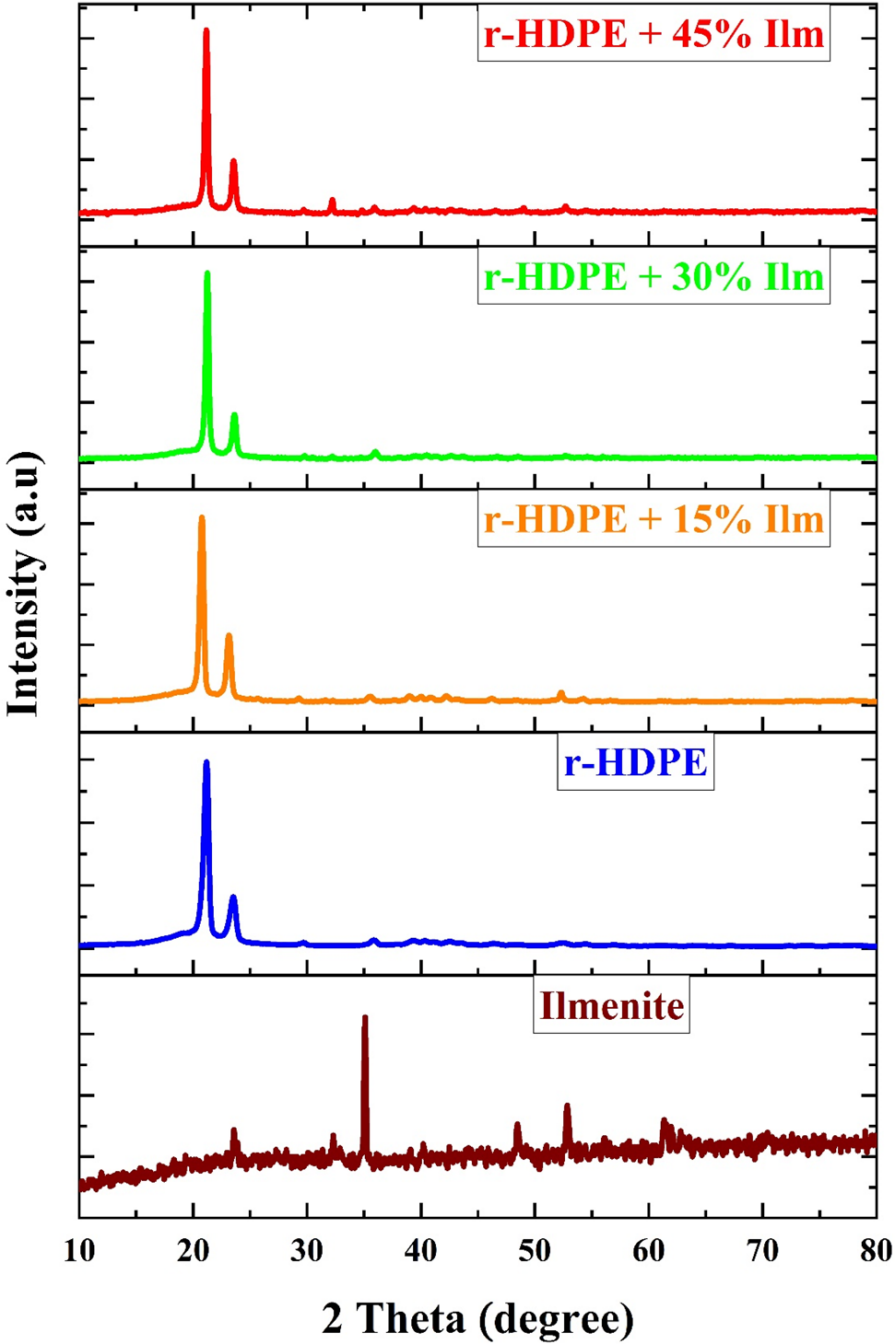
XRD patterns of r-HDPE + *x*% Ilm composite sheets.

Fig. 4 shows X-ray diffraction XRD patterns of ilmenite and r-HDPE + *x*% Ilm composite sheets. The XRD pattern of ilmenite indicates the existence of a single phase of pure ilmenite without any associated impurity structures. The figure shows that the ilmenite has a peak of at 2*θ* = 23.58°, 32.28°, 35.09°, 48.48°, 53.14°, and 61.34° corresponding to the planes (012), (109), (110), (024), (116), and (124) confirm the FeTiO_3_ rhombohedral structure (JCPDS file no. 01-075-1211).^18^

The pattern of the pure r-HDPE contains two strong reflection peaks at 2*θ* = 21.18° and 23.54°. Also, a gradual reduction in the intensity of the peaks of r-HDPE was observed with an increase in the ilmenite content. This suggests that the addition of ilmenite has an impact on the crystallinity of r-HDPE and may result in phase separation at higher ilmenite concentrations. In addition, the XRD patterns reveal no ilmenite peaks, indicating that the ilmenite powder is dispersed uniformly throughout the r-HDPE, besides the r-HDPE that serves as a protective layer to powder. The X-ray diffraction analysis confirmed the successful preparation of composite sheets comprising r-HDPE and *x*% Ilm.^19^

**Fig. 5:**
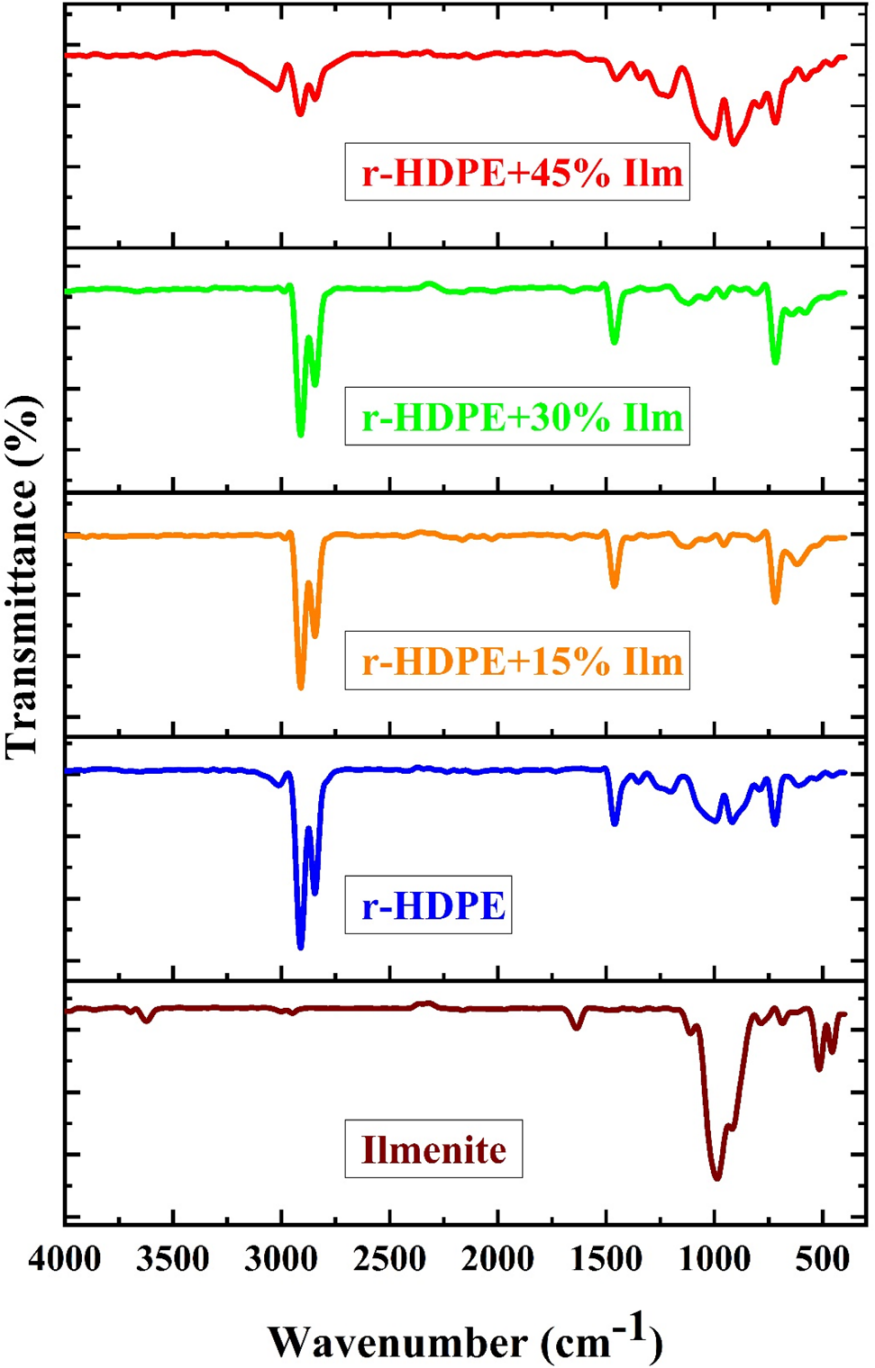
FTIR spectra of r-HDPE + *x*% Ilm composite sheets.

The functional groups of the ilmenite, pure r-HDPE, and r-HDPE + *x*% Ilm composite sheets were assessed *via* FTIR analysis, as presented in Fig. 5. For ilmenite ore, the peaks between 500 and 880 cm^−1^ correspond to Ti–O–Ti and Fe–O bonds, whereas the peak at 685.75 cm^−1^ is related to the characteristic FTIR peaks of ilmenite.^20–22^ The pure r-HDPE, as shown in Fig. 5, has two notable peaks at 2914.12 and 2845.33 cm^−1^, which belong to C–H symmetric and asymmetric stretching modes, respectively. Two further moderate C–H bending vibration peaks were observed at 1459.29 and 717.56 cm^−1^, which are associated with polymeric compounds containing more than four C atoms in the polymer chain. The wide band suggests the existence of hydroxyl groups and bound O–H stretching vibrations at ∼3300 cm^−1^. The existence of C–O stretching vibrations might explain the weak band at 1162 cm^−1^.^6,23^ Further, as can be observed, the r-HDPE + *x*% Ilm composite sheets has several new peaks of filler function groups, demonstrating the chemical grafting of ilmenite onto the r-HDPE backbone. Lastly, for the r-HDPE + *x*% Ilm composite sheets, the high bands of r-HDPE were shifted to lower wavenumbers, showing the interaction of ilmenite particles with r-HDPE, bonding Ti–O–Ti, and Ti–O–C.^22^

### 3.2. Mechanical properties of r-HDPE + *x*% Ilm composite sheets

**Fig. 6:**
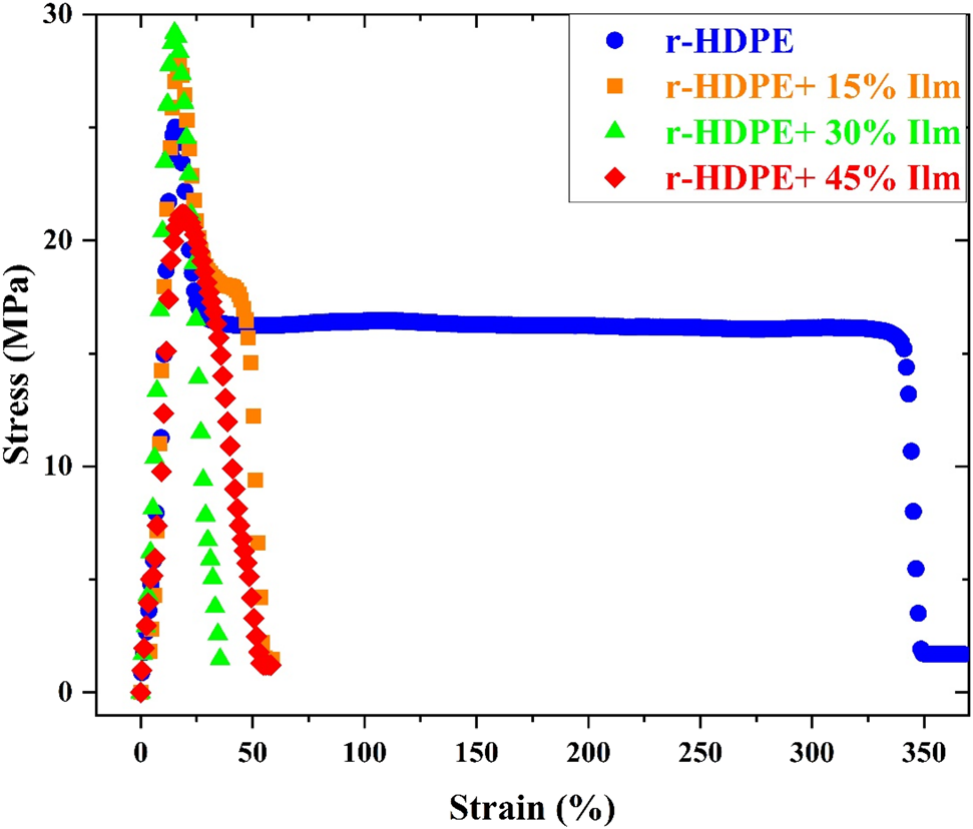
Typical tensile stress–strain curves of r-HDPE and r-HDPE + *x*% Ilm composite sheets.

The homogeneous distribution of inorganic components will considerably improve the mechanical characteristics of r-HDPE. The significance of examining the stress–strain relationship is to comprehend the new characteristics of the prepared composite and whether or not the loaded ilmenite would improve the mechanical properties of the composite. The stress–strain relationship in polymers is considered complicated and nonlinear in nature. Fig. 6 shows typical stress–strain curves of pure r-HDPE and r-HDPE + *x*% Ilm composite sheets with varying concentrations determined at room temperature and a constant loading rate (500 mm min^−1^).^24,25^

**Fig. 7:**
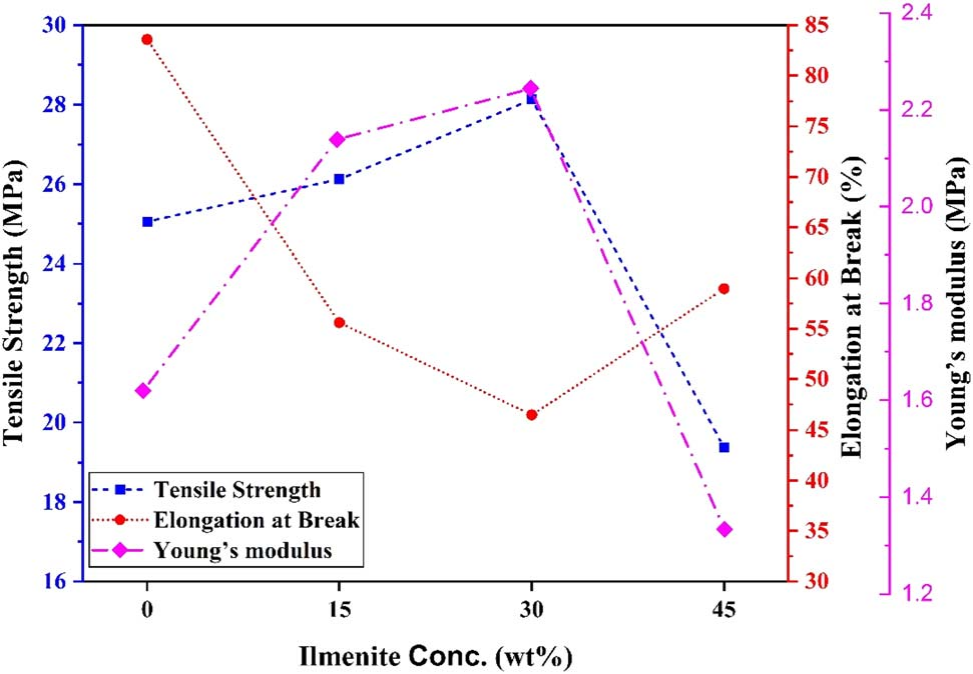
Mechanical parameters of r-HDPE + *x*% Ilm composite sheets.

To explore the influence of dopant ilmenite concentration on the mechanical characteristics of r-HDPE, the tensile strength (MPa), elongation at break percent, and Young's modulus (MPa) of r-HDPE + *x*% Ilm composite sheets were investigated (Fig. 7).^19^ According to Fig. 7, pure r-HDPE exhibits the stress–strain characteristic of a typically ductile material, with a tensile strength of 25.05 MPa and an elongation at a break of up to 83.56%. In the pure r-HDPE sample, a neck forms, and the same necking behavior was also seen in the 15 wt% ilmenite reinforced r-HDPE sample, although with lower percentage elongations.

Moreover, adding ilmenite with various concentrations into the r-HDPE matrix reduces composite ductility, causing the composites to become essentially brittle and break with substantially lower strain. In other words, by increasing the ilmenite content, reinforced r-HDPE composite sheets become stiffer than pure r-HDPE. This is because ilmenite is substantially stiffer than the r-HDPE and has a lower deformation capacity, resulting in a decrease in r-HDPE matrix strain overall.^24^ Interfacial delamination is one of the potential failure mechanisms for composite materials composed of a filler and a polymer matrix. Interfacial delamination takes place when it results in a loss of adhesion or bonding between the filler particles and the surrounding polymer matrix, resulting in separation or debonding at the interface. The fracture mechanics approach to tackling the issue of delamination in composite materials is based on the theory of anisotropic laminate elasticity.^26^ This theory analyzes the propagation of delamination fractures by taking the anisotropic nature of composite materials and their layered structure into account. An improved understanding of composite materials' failure mechanisms and vulnerable points is frequently associated with regions of high-stress concentrations, including defects, voids, or construction discontinuities, where fractures initiate and propagate according to the stressed conditions.^27^ Defects, voids, or structural discontinuities are regions of high-stress concentrations in composite materials with a filler and polymer matrix,^27,28^ where fractures can initiate and propagate under applied stress.

The tensile strength and Young's modulus rise significantly with increasing ilmenite content, as seen in Fig. 7, up to an optimal concentration of 30%, over which these parameters tend to decline. The composites' improved tensile strength and Young's modulus indicated the high ilmenite particle dispersion and interface interaction with the r-HDPE polymer matrix.^24,29^

### 3.3. Radiation shielding properties

Utilizing Phy-X/PSD, the shielding characteristics of r-HDPE + *x*% Ilm composite sheets with energies ranging between 0.015 and 15 MeV have been derived.

**Fig. 8:**
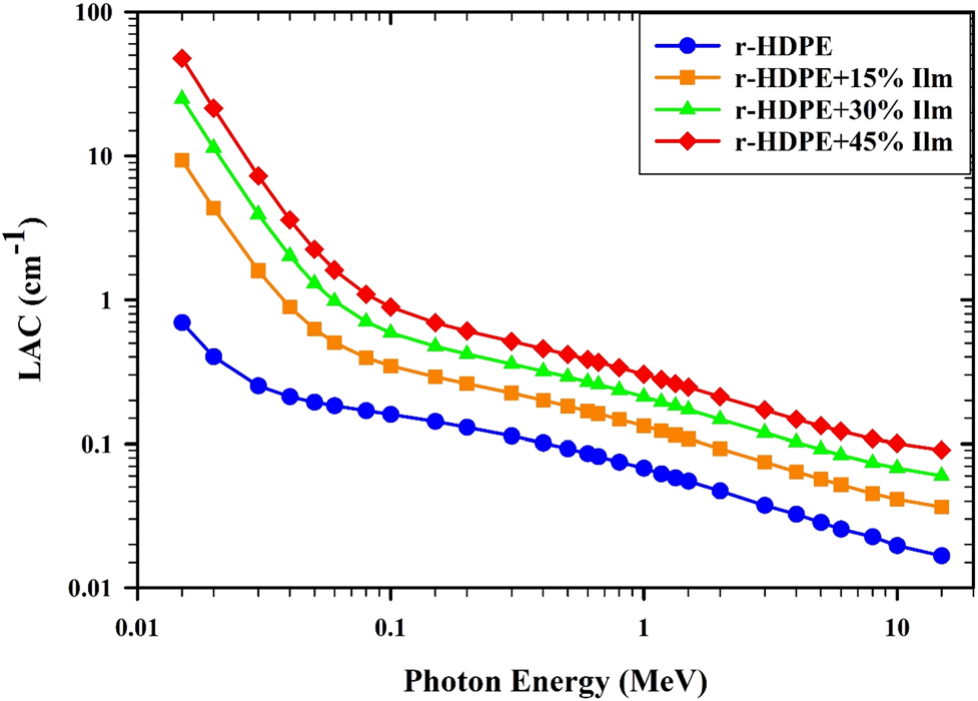
The relationship between the ilmenite content and the LAC of composite sheets.

**Fig. 9:**
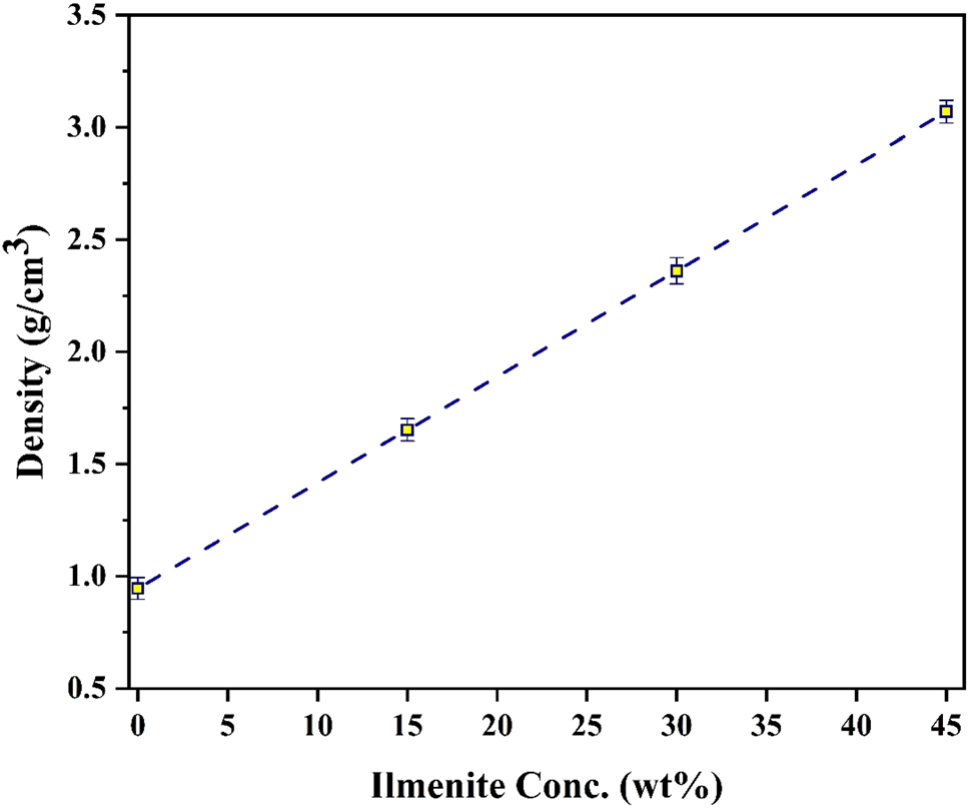
The density values of r-HDPE + *x*% Ilm composite sheets.

Fig. 8 depicts the variation of LAC values for r-HDPE composite sheets in the presence of incoming photon energy. At low energy (*E* < 0.5 MeV), the photo-electric effect (PE) interaction becomes particularly prominent, and the absorption cross-section is proportional to the atomic numbers *Z*^4^ or *Z*^5^ of the atoms. Therefore, the r-HDPE + 45% Ilm composite sheet offers the highest LAC values, while the pure r-HDPE has the lowest. The variance between LAC values of r-HDPE + *x*% Ilm composite sheets lessens as photon energy increases. This phenomenon can be attributed to the dominant influence of the Compton scattering (CS) interaction within the photon energy range of 0.5 < *E* < 1.5 MeV. The absorption cross-section is directly proportional to the atoms' atomic numbers (*Z*) in the r-HDPE + *x*% Ilm composite sheets. In the same context, the pair production (PP) interaction dominates at photon energies over 1.0 MeV.^16,17^ Also, the composite sheets of r-HDPE and *x*% Ilm significantly increased in molecular weight and density. The molecular weight and density of the sample have improved due to the addition of components that exhibit a gamma-ray absorption impact (such as Fe_2_O_3_, TiO_2_, Al_2_O_3_, SiO_2_, Al_2_O_3_, SiO_2_, and CaO).^30–32^ The investigated r-HDPE + *x*% Ilm composite sheets get denser with increasing ilmenite concentration. The observed rise in the density of r-HDPE + *x*% Ilm composite sheets may be ascribed to the density disparity between ilmenite, which has a density of 4.72 g cm^−3^, and r-HDPE, which has a density of 0.945 g cm^−3^. The density of r-HDPE + *x*% Ilm composite sheets was 0.945–3.069 g cm^3^; see Fig. 9.

**Fig. 10:**
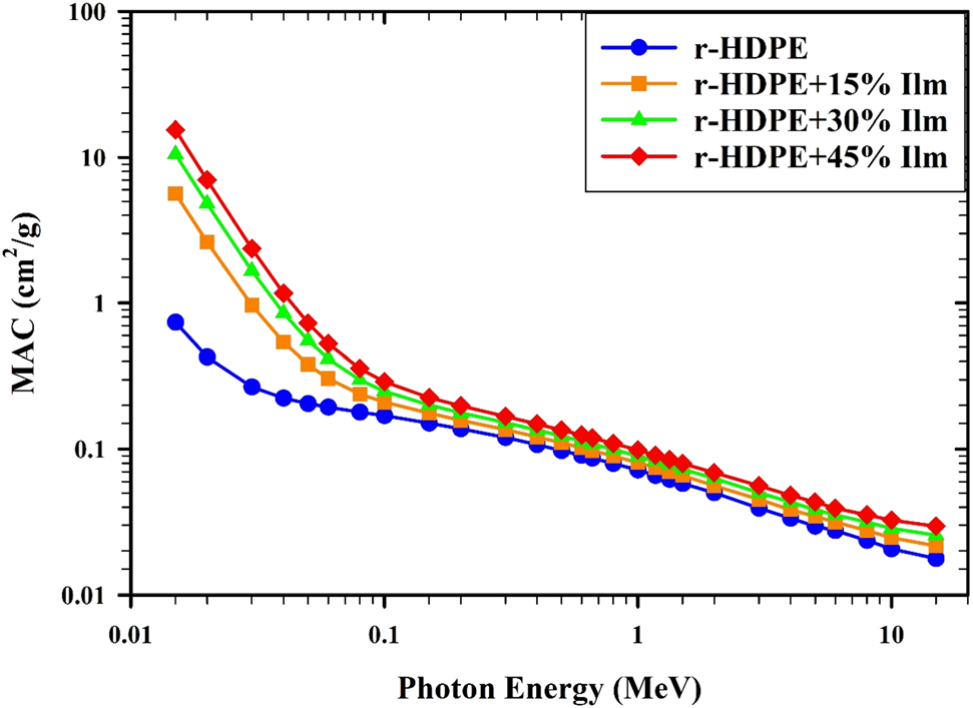
The relation between MAC and the energy of photons for r-HDPE + *x*% Ilm composite sheets.

The theoretical results of the mass attenuation coefficient (MAC) of r-HDPE + *x*% Ilm composite sheets are presented in Fig. 10. As the photon energy increases (*E* < 0.5 MeV), the MAC values decline rapidly. The photo-electric effect in the energy range is responsible for this behavior. As the photon energy increases up to 0.5 MeV, the MAC findings vary relatively slowly as the photon energy increases. This is due to the interaction of Compton scattering. As the ilmenite concentration enhances, so do the MAC results. Table 1 presents a comparative analysis of the mass attenuation coefficient values obtained through the Phy-X/PSD program and those obtained from XCOM. The MAC findings of r-HDPE + *x*% Ilm composite sheets generally decrease with increasing *E* and rise with increasing ilmenite concentration from 0 to 45% wt.

**Table 1:** Investigating the mass attenuation coefficients of r-HDPE + *x*% Ilm composite sheets

Mass attenuation coefficient (cm^2^ g^−1^)								
Energy (MeV)	r-HDPE		r-HDPE + 15% Ilm		r-HDPE + 30% Ilm		r-HDPE + 45% Ilm	
	XCOM	Phy-X	XCOM	Phy-X	XCOM	Phy-X	XCOM	Phy-X
0.015	0.74550	0.74549	5.68200	5.68241	10.61850	10.61933	15.55500	15.55626
0.02	0.43160	0.43155	2.64110	2.64095	4.85060	4.85035	7.06010	7.05974
0.03	0.27070	0.27066	0.97540	0.97534	1.68010	1.68001	2.38480	2.38469
0.04	0.22750	0.22750	0.54490	0.54488	0.86230	0.86226	1.17970	1.17964
0.05	0.20840	0.20841	0.38405	0.38403	0.55970	0.55965	0.73535	0.73527
0.06	0.19700	0.19698	0.30884	0.30882	0.42068	0.42065	0.53252	0.53249
0.08	0.18230	0.18229	0.24232	0.24230	0.30233	0.30230	0.36235	0.36231
0.1	0.17190	0.17192	0.21267	0.21268	0.25344	0.25345	0.29421	0.29421
0.15	0.15340	0.15342	0.17851	0.17852	0.20362	0.20363	0.22873	0.22874
0.2	0.14020	0.14018	0.16035	0.16034	0.18049	0.18049	0.20064	0.20064
0.3	0.12170	0.12168	0.13778	0.13776	0.15386	0.15384	0.16994	0.16992
0.4	0.10900	0.10895	0.12303	0.12298	0.13706	0.13701	0.15108	0.15104
0.5	0.09947	0.09947	0.11214	0.11213	0.12480	0.12480	0.13747	0.13747
0.6	0.09198	0.09198	0.10363	0.10363	0.11528	0.11528	0.12692	0.12692
0.6617	0.08809	0.08809	0.09922	0.09922	0.11035	0.11035	0.12148	0.12148
0.8	0.08078	0.08078	0.09095	0.09095	0.10113	0.10112	0.11130	0.11129
1	0.07263	0.07263	0.08175	0.08175	0.09087	0.09087	0.09999	0.09999
1.173	0.06711	0.06711	0.07553	0.07552	0.08394	0.08394	0.09236	0.09236
1.333	0.06284	0.06284	0.07073	0.07072	0.07861	0.07861	0.08650	0.08650
1.5	0.05910	0.05910	0.06653	0.06653	0.07396	0.07396	0.08139	0.08139
2	0.05064	0.05064	0.05709	0.05709	0.06354	0.06354	0.06999	0.06999
3	0.04045	0.04045	0.04582	0.04582	0.05119	0.05119	0.05656	0.05656
4	0.03444	0.03444	0.03924	0.03924	0.04404	0.04404	0.04884	0.04884
5	0.03045	0.03045	0.03492	0.03491	0.03938	0.03938	0.04385	0.04385
6	0.02761	0.02761	0.03187	0.03186	0.03612	0.03612	0.04038	0.04037
8	0.02383	0.02383	0.02786	0.02786	0.03189	0.03190	0.03593	0.03593
10	0.02145	0.02145	0.02539	0.02539	0.02932	0.02933	0.03326	0.03326
15	0.01819	0.01819	0.02209	0.02209	0.02599	0.02599	0.02989	0.02989

Further, a high agreement between the XCOM findings and the Phy-X/PSD values was observed. Compared to pure r-HDPE (MAC = 0.08809 cm^2^ g^−1^), the MAC values for r-HDPE + 45% Ilm composite sheet increased to 0.12148 cm^2^ g^−1^ at 0.662 MeV. An increase in the HDPE system's high-density ilmenite content could be responsible for this observed change. The prepared r-HDPE + *x*% Ilm composite sheets have better-shielding characteristics since more ilmenite has been incorporated into the r-HDPE matrix.

The capacity of a substance to absorb gamma radiation can be expressed in half value layer (HVL), mean free path (MFP), and tenth value layer (TVL) measurements. Reduced values of them are indicative of enhanced radiation absorption effectiveness.

**Fig. 11:**
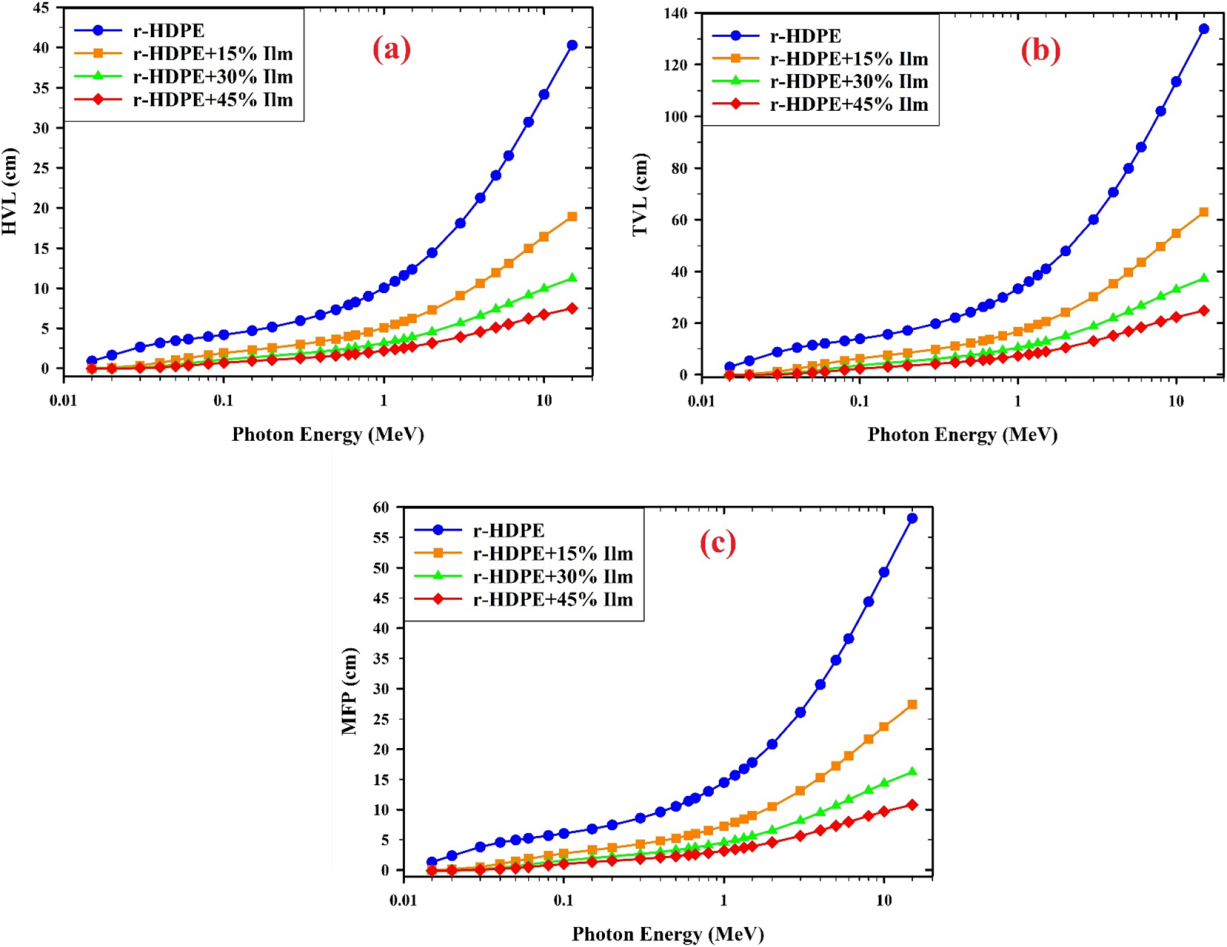
The varying values of (a) half-value layer (HVL), (b) tenth-value layer (TVL), and (c) mean free path (MFP) for composite sheets based-r-HDPE and *x*% Ilm.

**Fig. 12:**
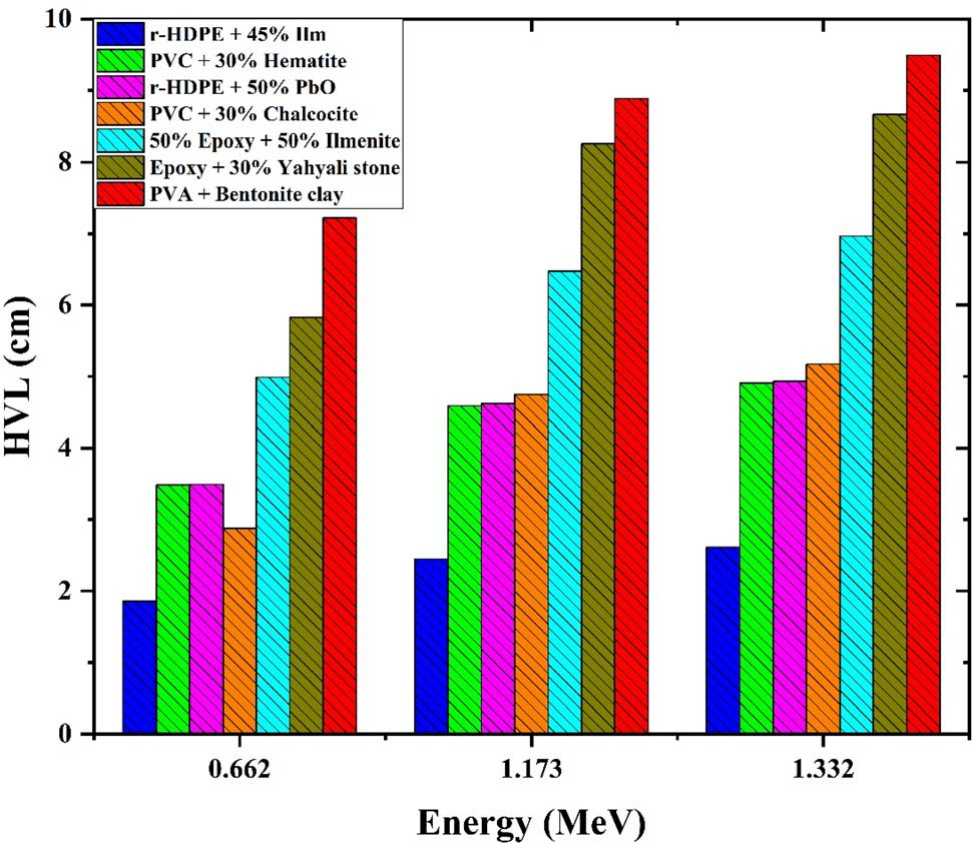
HVL as a function of photon energy for the r-HDPE + 45% Ilm composite sheet in comparison to the recently published studies.

Fig. 11(a) depicts the variation of HVL values with photon energy for all r-HDPE + *x*% Ilm composite sheets examined. It has been found that the HVL values are enhanced as photon energy increases in the 0.015–1 MeV range. HVL values in the 0.015–0.1 MeV range do not exceed 1 cm. Compared to the other composite sheets investigated, the r-HDPE + 45% Ilm composite sheets with the highest ilmenite concentration had the lowest HVL value. The HVL data of r-HDPE + *x*% Ilm composite sheets were documented at 1.332 MeV (0.662 MeV), with 11.673 (8.326), 5.929 (4.226), 3.735 (2.66), and 2.611 (1.859) cm for *x* = 0, 15, 30, and 45%, respectively. As a result, it proves more effective in absorbing radiation. In addition, Fig. 12 shows a variation of HVL as a function of photon energy for the r-HDPE + 45% Ilm composite sheet in comparison to the recently published studies. The HVL measurements obtained from the reported r-HDPE + 45% Ilm composite sheet (HVL = 2.611 cm), when exposed to 1.332 MeV, exhibit a lower magnitude compared to the HVL values of the epoxy + 30% Yahyali stone composite (9 cm);^33^ PVC + 30% hematite (4.9036 cm), +30% chalcocite (4.9303 cm);^34^ PVA + bentonite clay (8.6625 cm);^35^ 50% epoxy + 50% ilmenite composite (6.96631),^11^ and r-HDPE + 50% PbO (5.1716 cm).^6^

Fig. 11(b) displays the relationship between TVL values and incoming photon energy for the r-HDPE + *x*% Ilm composite sheets; we found that the TVL values rise as the incident photon's energy increases up to 1.5 MeV and that the link ceases to exist beyond this point. As ilmenite content is enhanced (from 0% to 45% wt), TVL values reduce.

The findings in Fig. 11(c) suggest that the Mean Free Path (MFP) values exhibit a dependence on photon energy until approximately 1.5 MeV, beyond which their correlation becomes unassociated.

Adding ilmenite to r-HDPE (0–45% wt) increases the effectiveness of r-HDPE sheets against gamma radiation across a wide range of photon energies, along with a reduction in mean free path values.

**Fig. 13:**
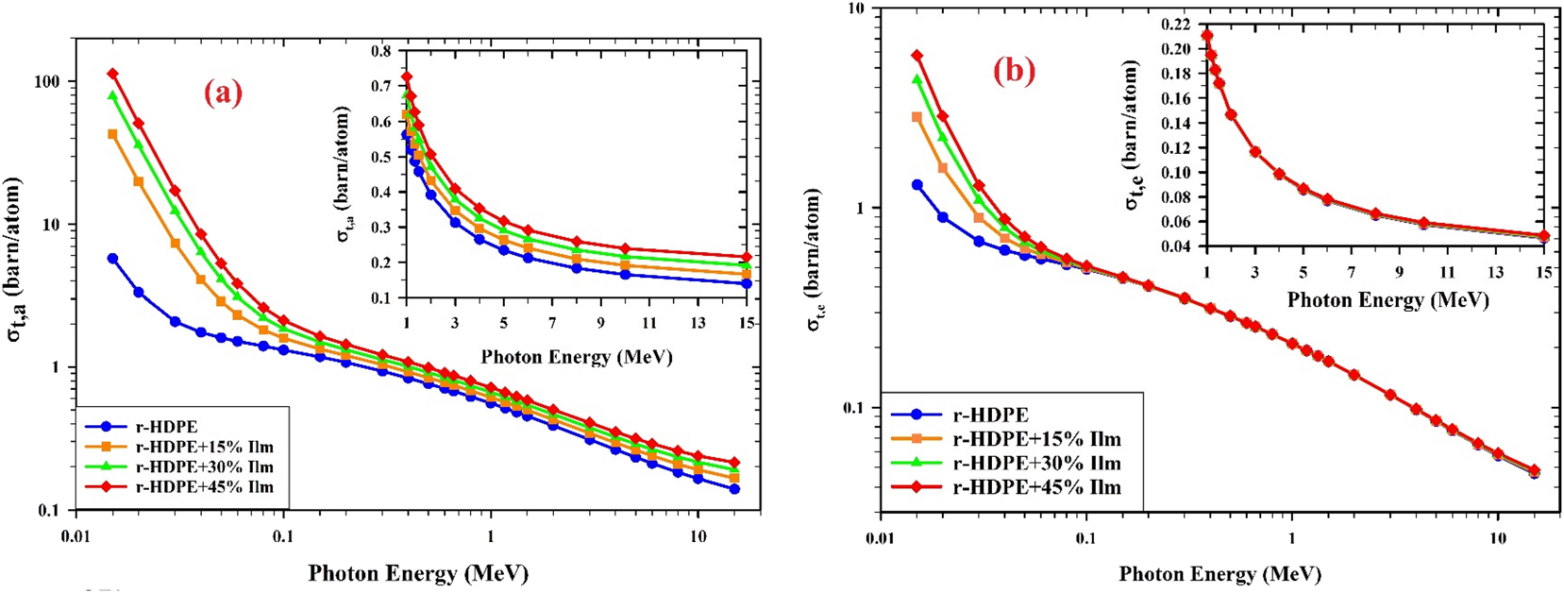
Variation (a) the total atomic cross section (*σ*_t,a_), (b) the total electronic cross section (*σ*_t,e_) *versus* the photon energy for r-HDPE + *x*% Ilm composite sheets.

The total atomic cross-section (*σ*_t,a_) as a function of gamma-ray energy for r-HDPE + *x*% Ilm composite sheets is shown in Fig. 13(a). As energy is increased, the total atomic cross-sections are significantly reduced. The photo-electric atomic cross-sections (*σ*_photo_) at low energy and the Compton scattering (*σ*_Compton_) at high energy are responsible for these notable differences. Fig. 13(b) displays the dependence of the total electronic cross section (*σ*_t,e_) on gamma-ray energies for the prepared r-HDPE + *x*% Ilm composite sheets; this gives the same behavior as the (*σ*_t,a_) and the MAC; namely, a decrease with increasing photon energy and an increase with increasing ilmenite content.^15^

The interpretation of the effective atomic number (*Z*_eff_) cannot be simplified to a singular value as it can vary based on the specific mechanism of photon interaction. The information presented pertains to the well-informed knowledge concerning materials interacting with gamma-ray photons;^16,36^6
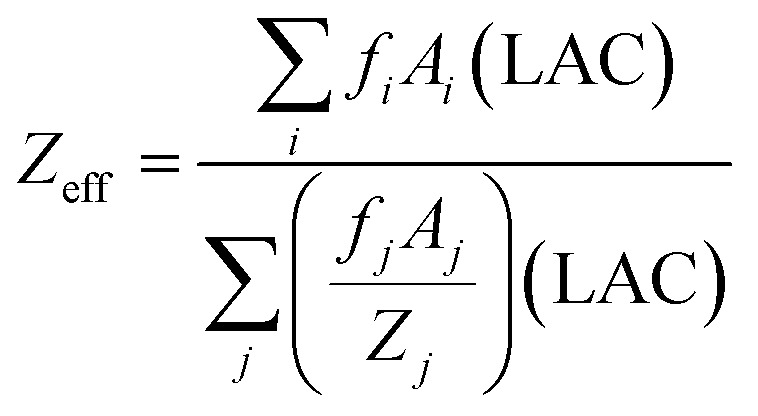


**Fig. 14:**
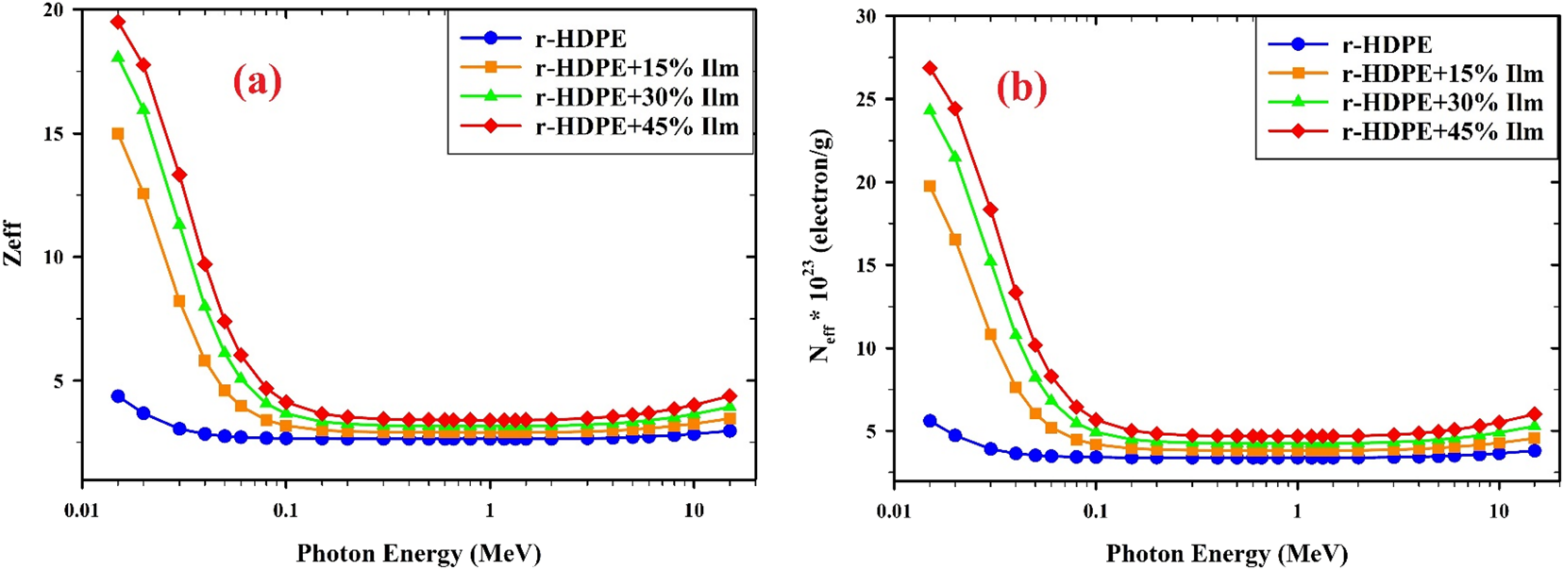
(a) The effective atomic number (*Z*_eff_) and (b) effective electron density (*N*_eff_) for r-HDPE + *x*% Ilm composite sheets.

Incorporating different elements in the manufactured r-HDPE composite sheets results in significantly varying values for the effective atomic number (*Z*_eff_). As shown in Fig. 14(a), the *Z*_eff_ of the r-HDPE + *x*% Ilm composite sheets increases when ilmenite is incorporated into the r-HDPE polymer. The photo-electric effect, the Compton scattering effect, and the pair production process all had interaction probabilities that were proportional to (*Z*_eff_)^4–5^, (*Z*_eff_), and (*Z*_eff_)^2^, respectively. As shown in Fig. 14(a), the increase in ilmenite concentration results in a rise in *Z*_eff_ owing to incorporating elements with relatively high atomic numbers (*Z* = 26 for Fe and *Z* = 22 for Ti). The energy dependence of *Z*_eff_ also exhibits behavior similar to that of MAC.^15,37^

The estimation of *N*_eff_, which refers to the effective electron density, can be achieved by utilizing the following equation;^38^7
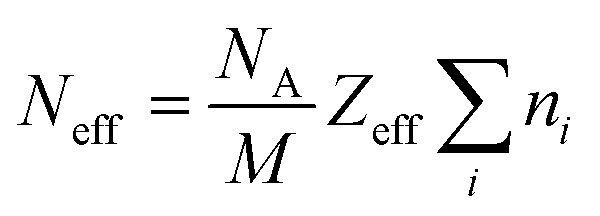


Fig. 14(b) illustrates the correlation between the ilmenite content and the effective electron number (*N*_eff_) for r-HDPE + *x*% Ilm composite sheets. Moreover, like *Z*_eff_, *N*_eff_ exhibited a similar response to the rise of ilmenite concentration. The composite sheet of r-HDPE and 45% Ilm demonstrates an equilibrium of nearly constant behavior. The observed behavior in *N*_eff_ of the r-HDPE + *x*% Ilm composite sheets can be attributed to the similarity in atomic numbers of the constituent polymers, primarily C, H, and O elements.^19^

## 4. Conclusion

This paper presents new recycled composite sheets of high-density polyethylene plastic and ilmenite mineral (Ilm) (0, 15, 30, and 45 wt%) as sustainable and flexible radiation shielding material. The ilmenite mineral was employed to enhance various r-HDPE attenuation and density features. The structure of composite sheets was evaluated using XRD, SEM, EDX, and FTIR. Moreover, adding ilmenite with various concentrations into the r-HDPE matrix reduces composite ductility. Also, the tensile strength and Young's modulus rise significantly with increasing ilmenite content. The study involved a comparison of the mass attenuation coefficient of composite sheets composed of r-HDPE and *x*% Ilm using both Phy-X/PSD and XCOM. The investigation explored the correlation between the ilmenite content and the *Z*_eff_ and *N*_eff_ for composite sheets. Overall, the r-HDPE + *x*% Ilm composite sheets' radiation-attenuating features make them suitable materials for radiation shielding applications.

## Author contributions

M. I. A. Abdel Maksoud: data curation, investigation, methodology, writing – original draft, writing – review & editing. Said M. Kassem: data curation, investigation, methodology, writing – original draft, writing – review & editing. A. H. Ashour: data curation, supervision. A. S. Awed: data curation, investigation, methodology, writing – original draft, writing – review & editing.

## Conflicts of interest

The authors declare that they have no conflict of interest.

## Supplementary Material
